# Mechanistic, Mathematical Model to Predict the Dynamics of Tissue Genesis in Bone Defects via Mechanical Feedback and Mediation of Biochemical Factors

**DOI:** 10.1371/journal.pcbi.1003604

**Published:** 2014-06-26

**Authors:** Shannon R. Moore, Gerald M. Saidel, Ulf Knothe, Melissa L. Knothe Tate

**Affiliations:** 1 Department of Biomedical Engineering, Case Western Reserve University, Cleveland, Ohio, United States of America; 2 Department of Orthopaedic Surgery, Cleveland Clinic, Cleveland, Ohio, United States of America; 3 Department of Mechanical & Aerospace Engineering, Case Western Reserve University, Cleveland, Ohio, United States of America; 4 Graduate School of Biomedical Engineering, University of New South Wales, Sydney, Australia; University of Pennsylvania, United States of America

## Abstract

The link between mechanics and biology in the generation and the adaptation of bone has been well studied in context of skeletal development and fracture healing. Yet, the prediction of tissue genesis within - and the spatiotemporal healing of - postnatal defects, necessitates a quantitative evaluation of mechano-biological interactions using experimental and clinical parameters. To address this current gap in knowledge, this study aims to develop a mechanistic mathematical model of tissue genesis using bone morphogenetic protein (BMP) to represent of a class of factors that may coordinate bone healing. Specifically, we developed a mechanistic, mathematical model to predict the dynamics of tissue genesis by periosteal progenitor cells within a long bone defect surrounded by periosteum and stabilized via an intramedullary nail. The emergent material properties and mechanical environment associated with nascent tissue genesis influence the strain stimulus sensed by progenitor cells within the periosteum. Using a mechanical finite element model, periosteal surface strains are predicted as a function of emergent, nascent tissue properties. Strains are then input to a mechanistic mathematical model, where mechanical regulation of BMP-2 production mediates rates of cellular proliferation, differentiation and tissue production, to predict healing outcomes. A parametric approach enables the spatial and temporal prediction of endochondral tissue regeneration, assessed as areas of cartilage and mineralized bone, as functions of radial distance from the periosteum and time. Comparing model results to histological outcomes from two previous studies of periosteum-mediated bone regeneration in a common ovine model, it was shown that mechanistic models incorporating mechanical feedback successfully predict patterns (spatial) and trends (temporal) of bone tissue regeneration. The novel model framework presented here integrates a mechanistic feedback system based on the mechanosensitivity of periosteal progenitor cells, which allows for modeling and prediction of tissue regeneration on multiple length and time scales. Through combination of computational, physical and engineering science approaches, the model platform provides a means to test new hypotheses *in silico* and to elucidate conditions conducive to endogenous tissue genesis. Next generation models will serve to unravel intrinsic differences in bone genesis by endochondral and intramembranous mechanisms.

## Author Summary

Arising as a consequence of trauma, tumor resection, removal of necrotic or infected tissue, and congenital abnormalities, critical-sized defects are too large to heal spontaneously and therefore require surgical intervention. New surgical approaches harness the regenerative power of the periosteum, a tissue membrane covering most bones, which provides a *niche* for stem cells and plays a key role in healing after injury. The interplay of mechanical, cellular and biochemical mechanisms involved in periosteum-mediated tissue genesis and healing remains elusive, providing the impetus for the current study. Here, we develop a mechanistic, mathematical model to predict the dynamics of tissue genesis by periosteum-derived stem cells within a bone defect surrounded by periosteum or a periosteum substitute. A mechanical finite element model is coupled with a model of cellular dynamics to simulate a tested clinical scenario in which the patient's own periosteum is left around the defect after injury. Model predictions incorporating mechanical feedback match spatiotemporal patterns of bone tissue regeneration observed in a series of *in vivo* ovine experiments. Through combination of computational, physical and engineering science approaches, the model platform provides a means to test new hypotheses *in silico*. This will provide criteria conducive to endogenous tissue genesis that can be tested in follow on experiments.

## Introduction

Critical-sized long bone defects pose a currently intractable challenge in orthopaedics as they do not heal spontaneously without surgical intervention and they are associated with significant disability and health care costs. Drawbacks of currently available treatment options, such as distraction osteogenesis, include long treatment durations, and soft tissue scarring. Alternative tissue engineering approaches offer a means to harness endogenous healing processes. A recently developed one-stage bone transport surgical technique [1], [2] capitalizes on the regenerative capacity of the periosteum, the membrane bounding all non-articular, outer bone surfaces. The periosteum provides rich vascular and nervous connections, as well as a niche for progenitor cell populations [3].

**Figure 1:**
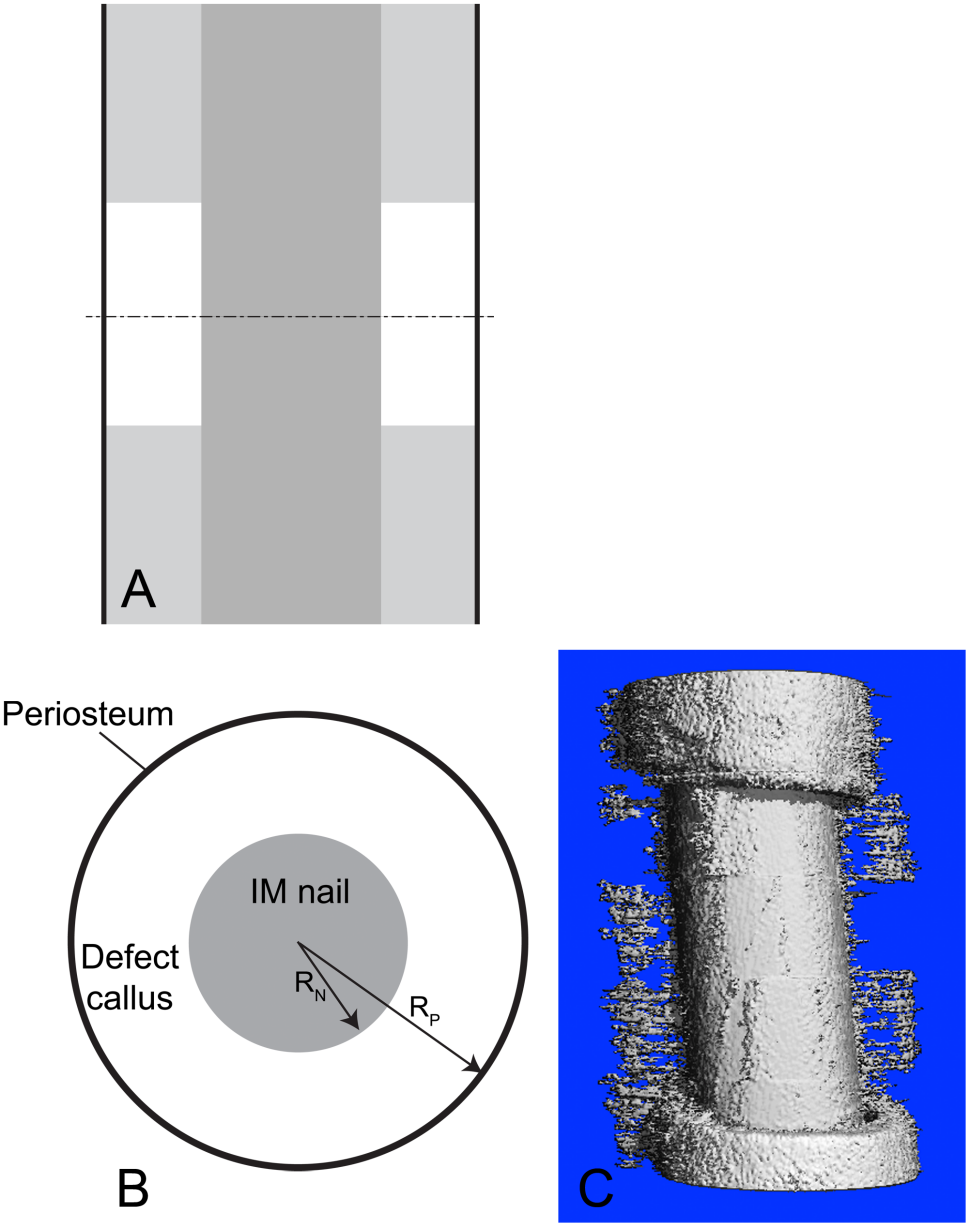
Prediction of bone tissue regeneration in a virtual model of a critical sized femoral defect tested in an ovine experimental model. (**A**) Schematic depiction of experimental model in longitudinal plane. Proximal (upper) and distal (lower) bone are represented in light gray, with the intramedullary nail in dark gray and the periosteum (lines) in black). The middle of the defect is indicated by the cut (dashed) line, giving the transverse orthogonal plane for the radial perspective (in **B**). (**B**) Spatial system diagram for the current model. System diagram depicting nascent tissue genesis (‘callus’) in the defect, defined as the region between the intramedullary (IM) nail and the surrounding periosteum. (**C**) Tissue genesis in the defect proceeds predominantly from the outside in, radially from the periosteum, rather than from the proximal and proximal and distal edges (longitudinally) toward the center of the defect, as evidenced by high resolution micro-CT of actual healing in the experimental ovine defect described by the predictive model [1], [4].

Briefly, the one-stage bone transport technique introduces a new defect, enveloped *in situ* by the periosteum, by osteotomizing the underlying cortical bone and transporting it distally into the original defect site (Fig. 1A, B). Tested in a 16-week ovine femoral defect model, bridging does not occur in absence of the periosteum (control group), which confirms the critical size of the defect. In contrast, all treated groups (periosteum ± bone graft) exhibit *de novo* bone tissue genesis within and bridging across the defect. Furthermore, infilling is facilitated in the absence of bone graft within the defect [1]. Using a similar *in vivo* ovine model, a follow on study was conducted to determine which periosteal factors (*e.g.* cells, periosteal strips) are essential for the observed periosteum-mediated defect healing. A periosteum substitute, designed such that desired factors can be placed in its pockets, is sutured around the defect [4]. Tissue genesis is rapid when periosteum derived cells (PDCs) seeded on collagen sheets or strips of periosteum with cells *in situ* are tucked into the pockets. These experiments demonstrate the power of PDCs to generate new bone *de novo* [4]–[10]. In addition, biochemical or molecular factors intrinsic to the periosteum enhance tissue genesis by PDCs even without a patent blood supply. Finally, periosteal strips tucked into the periosteum substitute result in infilling of the defect with less dense but a greater volume of tissue than vascularized periosteum *in situ* [4].

Further, bone regeneration and maintenance processes are intrinsically linked to mechanical environment. Phenomenological studies of bone regeneration have assessed the role of specific mechanical signals in regeneration dynamics and tissue formation, where magnitude and type of mechanical stimulus are mapped to a regenerated tissue phenotype [11], [12]. While these predictive models are capable of determining nascent tissue type locally, as a function of mechanical cues, the cellular and subcellular mechanisms of mechanically modulated tissue genesis are still not fully understood.

Recent studies with periosteum progenitor cells indicate their mechanosensitivity *in vitro* and *in situ*, with applied stretch, or tensile strain, resulting in upregulation of chondro- and osteogenic growth factors [5], [7], [13], [14]. While a variety of growth factors are implicated in the healing process, bone morphogenetic protein 2 (BMP-2) is widely involved in all stages of bone regeneration [8], [9], [15], [16]. Additionally, periosteal injuries heal predominantly via endochondral [17] and, less frequently via intramembranous [1], [3], ossification mechanisms, motivating a deeper understanding of the interplay of mechanical environment on BMP-2 production during periosteally mediated bone regeneration. Finally, defect healing, including initial tissue genesis and vascular perfusion 16 weeks after surgery, correlate to mechanical loading during the post-surgical healing period [18] as well as net change from baseline of the periosteum's mechanical environment [19].

A quantitative understanding of the endogenous and exogenous cues that facilitate tissue manufacture by resident progenitor cells requires an approach that bridges length scales of tissues (mm-cm), cells (µm) and molecules (nm) as well as time scales of tissue generation and healing (months), secretion of extracellular tissue matrix (ECM, days-weeks), and cellular processes (hrs-days) [3], [20]–[24],[25],[26]. Multi-scale mechanistic models that describe cellular-tissue dynamics provide a unique tool to un-/couple spatial and temporal effects or specific mechanical and/or biological effects. Model simulations predict the effect of parameters that affect system behavior, which can be tested experimentally. The continual interdigitation of simulations with experimental studies is the most efficient and least costly process by which we can make significant improvement in regeneration of large defects in bone [22], [23].

Previously developed mathematical models of bone regeneration have incorporated the processes of cell proliferation, differentiation and ECM secretion, as mediated by growth factor production but with parametric incorporation of mechanical stimuli [27]–[30]. In the current study, we develop a **mechanistic** model framework to predict the cellular, extracellular and mechanical progression of defect infilling, governed by the mechanically mediated production of BMP-2 by progenitor cells located in the periosteum. In this first generation model, bone morphogenetic protein (BMP) is chosen to represent of a class of factors that may coordinate bone healing. Of particular relevance to our labs' experience with a series of experiments using a common ovine critical sized defect model, periosteum (-substitute) mediated tissue genesis within the defect occurs predominantly in a radially inward fashion with no relation to distance along the defect from the proximal or distal edge [4]. Hence, we hypothesize that mechanoregulatory stimulation of progenitor cells located in the periosteum (OP, for osteochondroprogenitors [3]) can be used to predict tissue genesis in defects, measured as the area of *de novo* cartilage and bone (in cross section, Fig. 1A–C).

The novelty of the approach lies in the incorporation of a mechanistic model accounting for OP mechanical stimulation at the periosteal surface, with direct rather than parametric mediation by BMP-2 production representing a class of molecules mediating tissue genesis and healing. This enables us to model mechanical stimulation of the periosteum, driving OP cell proliferation and differentiation processes, which in turn result in defect infilling and concomitant stiffening of the callus, and which further provides a mechanism for mechanical feedback.

The following sections describe our experimental and computational modeling approach to characterize mechanical and biochemical factors related to healing of a bone defect. The defect separates two parts of the bone that are stabilized initially along the long bone axis by an interlocked intramedullary nail. Periosteum surrounds the defect and contains the OP cells, the ‘sources of healing’ which produce BMP and other factors that mediate bone healing (Fig. 1). With this model of cellular and tissue dynamics, incorporating mechanical and biochemical factors, simulations are presented that show the effects of each of the rate processes that contribute to tissue genesis and mineralization. Model predictions incorporating mechanical feedback match spatiotemporal patterns of bone tissue regeneration observed in a series of *in vivo* ovine experiments.

## Materials and Methods

### Mechanical Model to Estimate Stain Environment at the Periosteum

**Figure 2:**
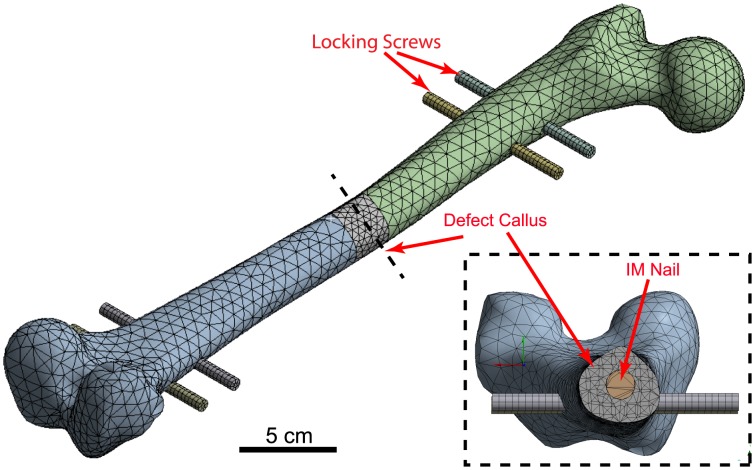
Set-up of mechanical finite element model. Simulation of the one-stage bone transport technique at the mid-diaphysis of a human femur, stabilized by an intramedullary (IM) nail and four locking screws.

A mechanical finite-element (FE) model of an adult human femur was established to approximate loading conditions at the surface of the periosteum during bone regeneration. Further, the FE model served as an input into a mechanistic mathematical model (**Development of a Cellular-Tissue Model**, below). The three-dimensional (3D) computer-aided design (CAD) geometry of the Sawbones standard femur model (third generation), created by M. Papini [31], was accessed online through the BEL Repository (https://www.biomedtown.org). The Sawbones femur represents a composite geometry, which has been validated experimentally as well as computationally to closely represent mechanical properties of the healthy femur [32]. Following import of the Sawbones model into a 3D CAD program (SolidWorks, Dessault Systèmes, Waltham, MA) the one-stage bone transport surgery was simulated on the model through the creation of a full 2.54 cm critical sized defect at the mid-diaphysis, measured as the midline between the femoral head and the condyles. The defect is stabilized with a stainless steel intramedullary (IM) nail of 35 cm length, 12 mm diameter, and interlocked to the proximal and distal femur via four locking bolts of 10 cm length and 7 mm diameter (Fig. 2).

Cancellous bone was not accounted for in the mechanical model, as it has been shown previously to alter predictions of strain by less than 1% in a similar linearly elastic model [33]. Joint contact forces, as well as the balancing iliotibial components of the abductors and *tensor fascia latae* were applied to represent the early stance phase [34], while maintaining the condyles in a fixed position. Meshing and FE analysis was performed (Ansys 14.5, Ansys, Inc. Canonsburg, PA), with a minimum of 150,000 quadratic tetrahedral elements.

The nascent tissue comprises extracellular matrix (ECM) in the form of rapid proliferative woven bone and/or osteochondral tissue in the process of ossification [3], [4], [35]. Tissue genesis proceeds *in vivo* within the defect throughout the healing process. At any point in time, the tissue (ECM) is idealized as either a cartilaginous and/or osseous template in the process of endochondral ossification. The periosteum is idealized as a membrane of negligible thickness relative to the scale of the defect site. The mechanical environment on the surface of the nascent tissue formed in the defect is therefore assumed to be the same as that of the comparatively soft and elastic periosteum. Material properties are applied based on commonly used values from published studies (Table 1). To assess and account for the evolving mechanical environment at the surface of the periosteum throughout tissue genesis and healing, the material properties of the nascent tissue (ECM, also referred to as callus) evolve over time with repeated simulations. Specifically, at 10 discrete intervals, representing phases of the defect infilling and healing process over time, the Young's modulus and Poisson's ratio are adjusted using mixture theory. The mechanical properties are defined, based on the state of the tissue, falling between the beginning and end states of the endochondral ossification process, with nascent tissue comprising 100% cartilage at one end and 100% cortical bone at the opposite end of the spectrum. The applied material properties are then calculated as a weighted average of the Young's modulus and Poisson's ratio.

**Table 1:** Material properties applied to the mechanical finite element model.

Material	Young's Modulus, E (GPa)	Poisson's Ratio, γ
**Cartilage**	0.01 [70]	0.167 [71]
**Cortical Bone**	17.0 [72]	0.325 [73]
**316 stainless steel**	193	0.3

**Figure 3:**
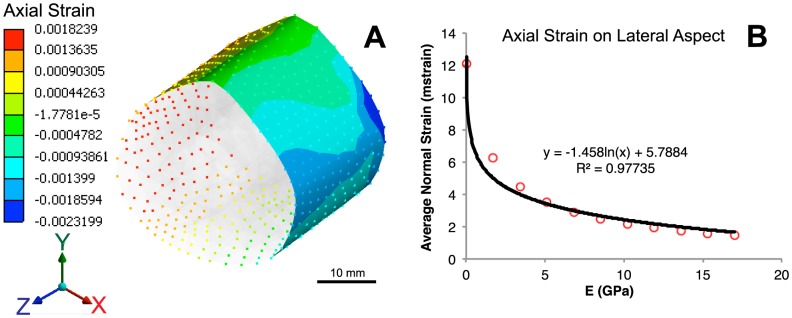
Relationship between nascent tissue material properties and axial strain. (**A**) Map of axial strains at the outer surface, representing periosteal mechanical environment. (**B**) Extracted average axial strain on the lateral aspect as a function of tissue modulus, fit with logarithmic relationship.

Following simulation with the described loading, boundary and material conditions, the strains at the surface of the defect callus surface are extracted (Fig. 3) as inputs for the **Cellular-Tissue Model** (see below for details). In context of the current study, the axial strain, 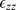, which represents the largest measure of normal strain by approximately an order of magnitude, is assessed from each simulation. Strains are recorded as a function of nascent tissue's material properties at the periosteal surface. Based on previous experimental strain mapping studies from our group, positive strains (tensile) are experienced on the lateral aspect of the femur, while negative strains (compressive) are experienced on the medial aspect [19]. For this first generation of the model, only the tensile, positive strains are assessed as they have been more thoroughly described in the literature. Axial strains 90° orthogonal to the lateral aspect are averaged to approximate a representative value, and plotted as a function of Young's modulus (Fig. 3). The further development of mathematical relationships describing the effect of strain on periosteal osteoprogenitor cell behavior is outlined under **Parameter Estimation and Simulation Strategy**.

### Development of a Cellular-Tissue Model

**Figure 4:**
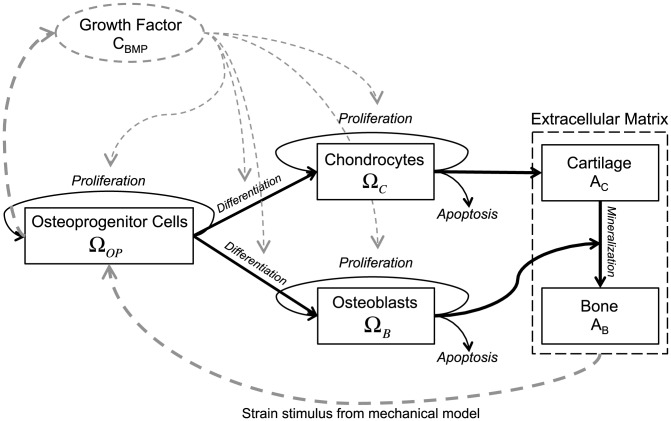
System diagram of cellular processes. Periosteally mediated bone regeneration following mechanical strain stimulus of progenitor cells located in the periosteum, mediated by expression of, *e.g.* BMP-2. For the purposes of the current model as a foundation for next generation models, BMP is an isolated representative factor implicated in regulating all of the described processes and as such represents a class of signaling molecules whose mechanistic roles can be probed explicitly in follow on studies.

A mechanistic, mathematical model is developed to quantify the dynamics of cellular and tissue components that can form in a bone defect surrounded by periosteum (depicted schematically in Fig. 1). Definition of a cylindrical coordinate system best depicts tissue genesis described the experimental model [4], analogous to the geometry of a critical sized defect in cross-section and in cognizance of the small length scale cell activity relative to the span of the defect. Furthermore, nascent periosteum derived cell-modulated bone genesis in critical sized defects enveloped *in situ* by either native, intact periosteum [1], [36] or periosteum substitute [4], [36] proceeds primarily from the outer radial boundary of the bone defect inwards rather than from the axial proximal and distal edges of the defect toward the middle of the defect length. In this model, the primary regulatory processes of BMP-2 are probed in context of bone tissue genesis via endochondral pathways. While BMP is chosen generally to represent a class of molecules that modulate tissue genesis and healing, BMP-2 exerts unique effects on osteoprogenitor cells, chondrocytes and osteoblasts. In overview, a mechanical feedback loop is established, where chondrocytes produce cartilaginous ECM (cartilage), which is subsequently mineralized into bone by osteoblasts. The process of endochondral ossification results in evolution of material properties during tissue genesis, effectively stiffening the defect site and decreasing the mechanical strain experienced at the bounding periosteal surface during the course of healing. Mechanosensistive osteoprogenitor cells within the periosteum upregulate BMP-2 production as a function of their prevailing mechanical environment (strain). A decrease in production of BMP-2 follows stiffening of the tissue regenerate. BMP-2 in turn regulates the cell processes of proliferation and differentiation (Fig. 4).

### Dynamics of Osteochondroprogenitor Cells

The osteochondroprogenitor (OP) cells located within the cambium layer of human periosteum are capable of differentiating along chondrogenic and osteogenic pathways [37]. BMP-2 is known to regulate key biological activities of periosteal OP cells. Human mesenchymal stem cells (MSCs, of which OPs are a subset [38]) proliferate significantly faster following BMP-2 treatment relative to untreated control cells [39]. Additionally, differentiation of periosteal progenitors into chondrogenic and osteogenic cells is regulated by BMP-2 in a dose-dependent manner [8], [16]. While some migration of OP cells may occur, a simplifying assumption of no migration is made in the current model iteration, as cell tracking experiments indicate that periosteal OPs remain close to the periosteal surface [17]. Future versions of the model will be developed to determine eventual roles of migration activity on healing.

The primary behavioral processes of OP cells comprise proliferation and differentiation into chondrocytes (C) or osteoblasts (B), while remaining close to the periosteum at 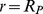. The OP number per unit surface area at the periosteum 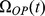 changes with time according to:
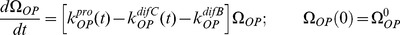
(1)Injury and mechanical stimulus of periosteum results in a rapid proliferation of OP cells [40]; proliferation and differentiation of OP cells serves to maintain a population of multipotent cells in the periosteum throughout healing. As long as the density of OP cells is below a critical density, the rate of OP cell proliferation follows a Monod relationship for a rate-limiting factor (BMP):
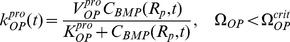
(2)When the density of OP cells is above the critical density, the rate of proliferation matches the rate of differentiation to chondrocytes and osteoblasts:
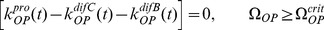
(3)such that population of OP cells remains constant in the periosteum during healing.

The rate of OP differentiation to chondrocytes or osteoblasts similarly follows a Monod relationship for BMP:
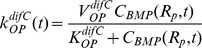
(4A)

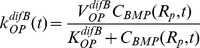
(4B)In the Monod relationship, V represents the maximum rate and K is the bound BMP concentration at V/2.

### Dynamics of Bone Morphogenetic Protein

As BMPs are the most well-known and researched musculoskeletal growth factors [41], they are the focus of the framework for growth factor activity in the model presented here (although future iterations of the model may be expanded to include an array of growth factors and cytokines that modulate tissue genesis and healing). BMPs are widely implicated as important regulatory factors during all stages of bone regeneration including cellular proliferation, differentiation, ECM production and apoptosis [42]. Recently, BMP-2 has also been shown to play an key role in periosteum-mediated bone regeneration [43], where deletion of BMP-2 postnatally almost completely blocks osteogenic and chondrogenic differentiation of periosteal progenitor cells [16]. The OP cells within the periosteum are mechanosensitive, with BMP-2 upregulation detectable within the periosteum *in vivo* as shortly as one hour after loading stimulation [44]. The periosteum also responds to mechanical stimulation by a robust proliferation of OP cells within the cambium layer [14], [45].

BMP-2 (here labeled as BMP for simplicity) is produced by mechanical stimulation of OP cells and diffuses away from the periosteum into the defect site. The system of BMP anatagonists is complex and not yet fully understood, but it appears to be a self-regulatory negative feedback loop [46]. To keep this aspect as straightforward as possible in the current generation of our model, we idealized deactivation of BMP from the system as a metabolic removal by cell uptake and consumption. As our understanding of the biology gains sophistication, the model will be refined to provide a more realistic reflection of the complex biological situation.

Hence, the number of BMP units per unit volume in the defect, 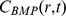, change according to:
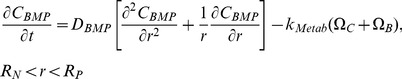
(5)Initially, no BMP is present: 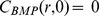. Furthermore, at the surface of the nail, BMP cannot penetrate:
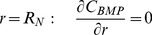
(6)At the periosteum, the rate of production of BMP, which is proportional to the number density of the OP cells, equals the BMP diffusion flux into the defect. The rate of production depends on the strain at the periosteal surface, modeled as the axial normal strain, 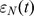:
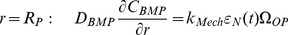
(7)where 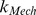 relates periosteal strain to BMP production.

### Mechanical Factors

The mean axial normal strain, 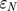 is calculated as an empirical function of the average elastic modulus 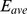: 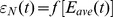 described in the **Parameter Estimation and Simulation Strategy** section. The average elastic modulus is integrated over the defect region:
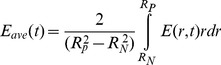
(8)The local elastic modulus depends on the area fractions of cartilage (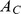) and bone (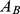), and is calculated using a law of mixtures, where the elastic modulus for cartilage (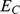) and for bone (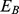) are known constants:
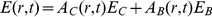
(9)and where the fraction of ECM at any position in the cross-section of the defect is:
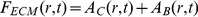
(10)


### Chondrocyte Dynamics

Chondrocytes (C) migrate according to random motility, proliferate, and die by apoptosis, where 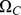 is the number of chondrocytes per unit volume:
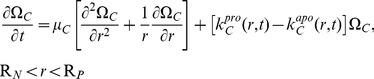
(11)The rate coefficient for proliferation depends on the local BMP concentration:
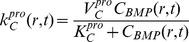
(12)Chondrocyte apoptosis occurs at a critical density of the local ECM, i.e. 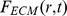:
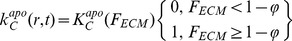
(13)Migrating cells do not enter into the intramedullary cavity (filled by a nail) so that the cell motility flux is zero; consequently,
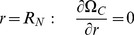
(14)Close to the periosteum, OP cells that differentiate into C cells migrate into the defect space. The rate of migration per unit surface area equals that of cell differentiation:
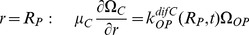
(15)Initially, no chondrocytes are present in the defect: 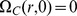


### Osteoblast Dynamics

Osteoblasts (B), which are formed by the differentiation of OP cells, migrate by random motility, proliferate and die by apoptosis. The number of osteoblasts per unit volume, 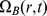, change with time and position as follows:
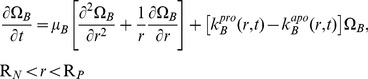
(16)The rate coefficient for proliferation depends on the local BMP concentration:
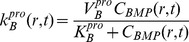
(17)Apoptosis of osteoblasts occurs when they are surrounded by a critical density of bone:
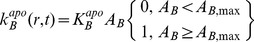
(18)Since migrating cells do not enter into the intramedullary cavity (filled by a nail), the cell motility flux is zero; consequently,
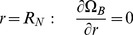
(19)Close to the periosteum, OP cells that differentiate into B cells migrate into the defect space. The rate of migration per unit surface area equals the rate of cell differentiation:
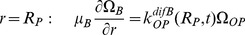
(20)Initially, no osteoblasts are present in the defect: 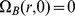


### Production of Extracellular Matrix

The extracellular matrix (ECM) consists of cartilage and mineralized bone. Cartilage is produced by chondrocytes, and is mineralized (transformed) into bone, mediated by osteoblasts. In any region of the defect (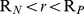), the ECM formation is considered to be characterized by *neighborhood area fractions* of cartilage (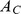) and bone (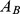), such that
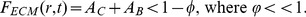
(21)


Within the defect (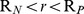), the local area fraction of cartilage increases in proportion to the local density of chondrocytes, and decreases in proportion to 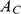 as the rate of mineralization:
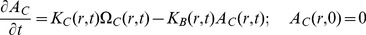
(22)The rate coefficient of cartilage formation 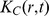 varies with 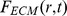, the local area fraction of total ECM. When 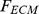 is small, the rate of production of cartilage is a maximum. When 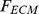 increases beyond a critical value, 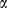, the rate slows as 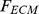 increases due to contact inhibition. Cartilage production stops when 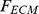 reaches a critical maximum density 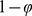:
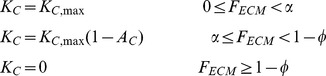



Mineralized bone is produced by osteoblasts mineralizing the cartilage template, the presence of which must precede bone formation. The local area fraction of mineralized bone increases in proportion to 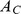:
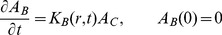
(23)The rate coefficient for bone mineralization, 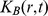, depends upon the local cell density of osteoblasts:
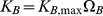



### Parameter Estimation and Simulation Strategy

Proliferative rates are estimated based on literature values for osteoprogenitor cells, chondrocytes and osteoblasts as: 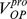 = 1.5 fold/day [47], 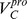 = 1.3 fold/day [48] and 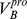 = 2.4 fold/day [49]. The diffusivity of BMP-2, 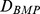, is approximated as the diffusivity of protein in cytoplasm: 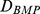 = 0.013 cm^2^/day [50]. The motility of osteoblasts and chondrocytes is estimated as one order of magnitude lower than that of BMP: 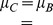
1.3×10^−3^ cm^2^/day. The maximal rate of cartilage and bone production per day by chondrocytes and osteoblasts are estimated as: 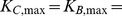
3×10^−6^ cm^2^/(cell day) [51], [52].

The mean axial normal strain is calculated as a function of 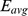 (GPa), which is determined from the average of 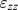 for all lateral nodes at the periosteal surface from finite element outputs:
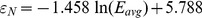
(24)where 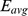 is in GPa (Pa^9^) and 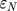 is in millistrain (ε^−3^).

To estimate 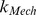, we consider a periosteal tensile strain of 2.5 millistrain, experienced at the lateral surface in a rat forelimb model [53] in context of strain magnitudes predicted on the corresponding surface of our current FE model. In the experimental rat model, the strain induces a four-fold upregulation of BMP production at the periosteal surface, where a one-fold increase is comparable to the non-loaded side. Although the alignment of the strain gage during measurements was not reported in this study, compressive and tensile strains are reported, and we assume that the strains represent axial components. Simply put, a 100% increase in BMP production represents a two-fold upregulation, and a 300% increase represents a four-fold increase in BMP production at the periosteal surface (*e.g.* 100 pg BMP increasing by 300% would be 100 plus 300 pg, resulting in 400 pg total or a four-fold increase). We then apply the experimental observations relating strain (2.5 millistrain or 0.25%) and upregulation of BMP production (300%) in the rat model [53] to our FE model, which predicts axial strains on the surface of the human femur to range from zero to a maximum of 12 millistrain or 1.2%, with most values on the order of magnitude of 0.25% (*per* method of calculation outlined in **Mechanical Model to Estimate Stain Environment at the Periosteum**). Hence, assuming a linear relationship between strain and BMP production, the following value of k_Mech_ is established, which represents the percent increase in BMP production with a given strain: 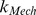 = 1.2 as a factor increase in BMP production over baseline per unit of microstrain.

The governing equations described previously are transformed to dimensionless versions (Fig. S1). Subsequently the spatial derivatives are discretized so that the model can be represented as an initial-value problem (Fig. S2). Numerical solution of this problem was obtained by applying a code for stiff differential equations “ode15s” in MATLAB R2011b (MathWorks, Natick, MA). For the first set of simulations, all dimensionless parameters are set to 1, except the calculated cell motilities, 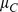 and 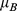 and the mechanical stimulus parameter, 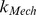. In subsequent simulations, parameters are varied independently to determine the relative effect on known outcome measures of ECM area fractions, 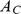 and 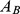.

## Results

Accounting for the experimentally observed, near complete infilling of the defect site with mineralized bone after 16 weeks of healing, a baseline of dimensionless parameters was established to describe the ideal healing state (ECM outcome) at 16 weeks. The model was used to predict mechanically mediated growth-factor concentration gradients, cell density dynamics, as well as ensuing tissue regeneration outcomes consistent with defect infilling.

**Figure 5:**
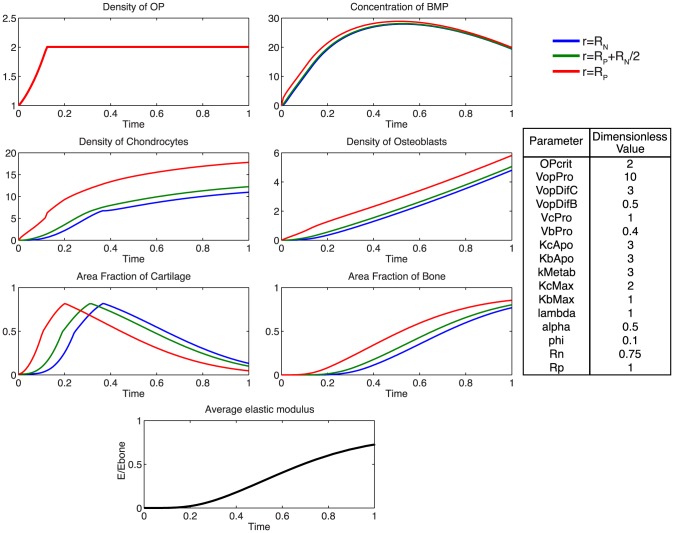
Parameter analysis for total defect infilling. Parameters optimized to achieve defect healing as a mineralization of the cartilage precursor template over the dimensionless time scale.

**Figure 6:**
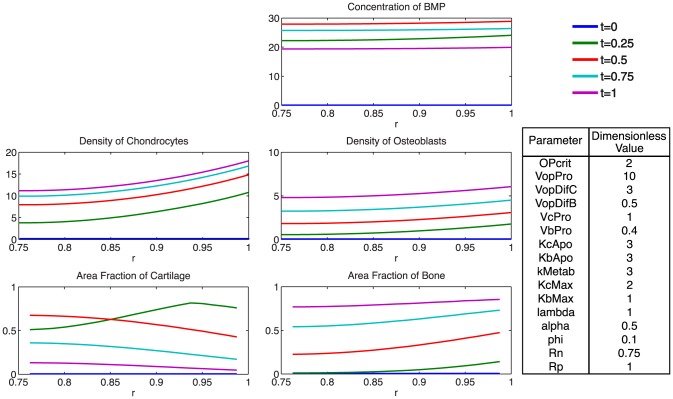
Spatial depiction of total tissue genesis for one representative set of parameter values, showing concentrations of BMP, chondrocytes, and osteoblasts, as well as area fractions of cartilage and bone as functions of radius at several time increments.

At the onset of healing, mechanical stimulation results in a rapid proliferation of osteoprogenitor cells within the periosteum, and an increase in BMP concentration (Fig. 5). The rapid diffusion of BMP from the periosteum to the intramedullary nail, relative to the expected total time course for tissue mineralization, results in a small spatial gradient of BMP (Fig. 6). Following increases in chondrocyte and osteoblast densities, metabolic consumption of BMP, coupled with decreased BMP production by osteoprogenitors via increasing nascent tissue stiffness, results in a gradual decrease in BMP concentration over time.

A rapid proliferation of osteoprogenitor cells within the periosteum is followed by saturation at the critical density, 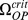. The relatively faster differentiation of chondrocytes from osteoprogenitor cells contributes to a large area of cartilage formation, mechanically stabilizing the defect at early time points, and providing a template for subsequent mineralization by osteoblasts. To achieve defect infilling in the model, chondrocyte proliferation must proceed at a faster rate than osteoblast proliferation; this differs from experimentally measured relative rates, which indicate two-fold faster proliferation rates of osteoblasts compared to chondrocytes [48], [49]. Inhibition of efficient nutrient diffusion due to tissue generation in the defect (increased ECM area fraction) is idealized to trigger apoptosis in the model [54]. Osteoblasts are assumed to be sensitive only to the surrounding fraction of mineralized tissue, as they actively convert cartilage to bone. The idealized representation of osteoblastic apoptosis in the model would likely be observed biologically as apoptosis or transformation to osteocytes, as a subset of osteoblasts become embedded in their surrounding mineralized matrix, and form a network of osteocytes, for nutrient exchange [55], [56]. Accounting for the idealized nature of the current model, it will be desirable to include explicit biochemical, cellular and environmental cues triggering apoptosis of chondrocytes and osteoblasts in next generation models [57], [58], [59].

Rapid chondrocyte proliferation results in early formation of an immature tissue template. ECM area fraction is higher in close proximity to the periosteum, attributable to the motility of chondrocytes into the defect space following differentiation from osteoprogenitor cells. The slowly increasing population of osteoblasts subsequently transforms the cartilage template into mineralized bone, at half the rate of cartilage production by chondrocytes. At the final time-point, approximately 80% of the tissue regenerate comprises *de novo* mineralized bone, which is reflected in the progressive increase in elastic modulus.

**Figure 7:**
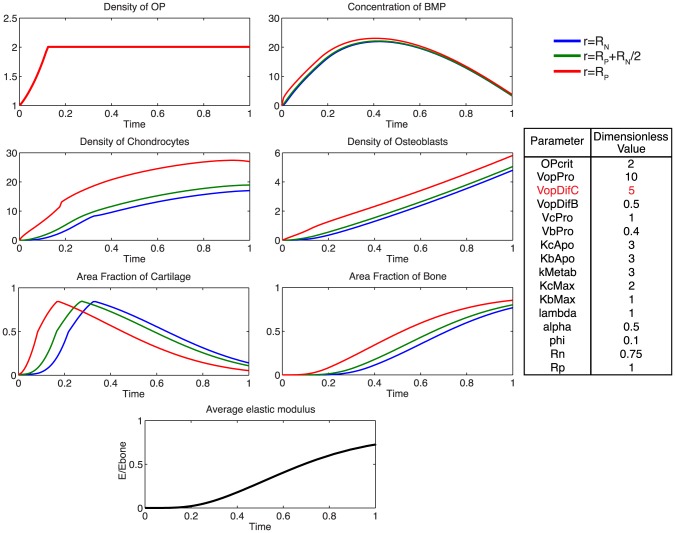
Effect of increasing 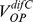. Increasing the rate of differentiation of progenitor cells to chondrocytes increases the density of chondrocytes and contributes to a more rapid consumption of BMP. Increased area fraction of cartilage is produced slightly sooner, indicative of more rapid tissue genesis.

**Figure 8:**
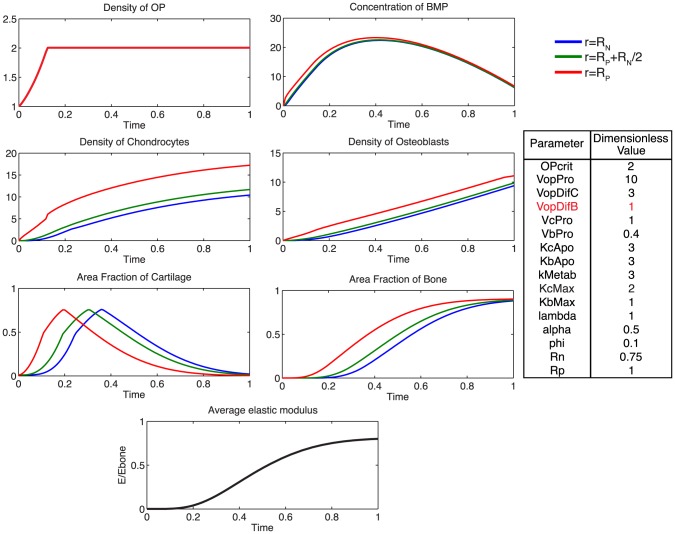
Effect of increasing 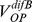. Increasing the rate of progenitor differentiation into osteoblasts results in a greater density of osteoblasts, more rapid consumption of BMP, and a more rapid mineralization of cartilage to bone.

**Figure 9:**
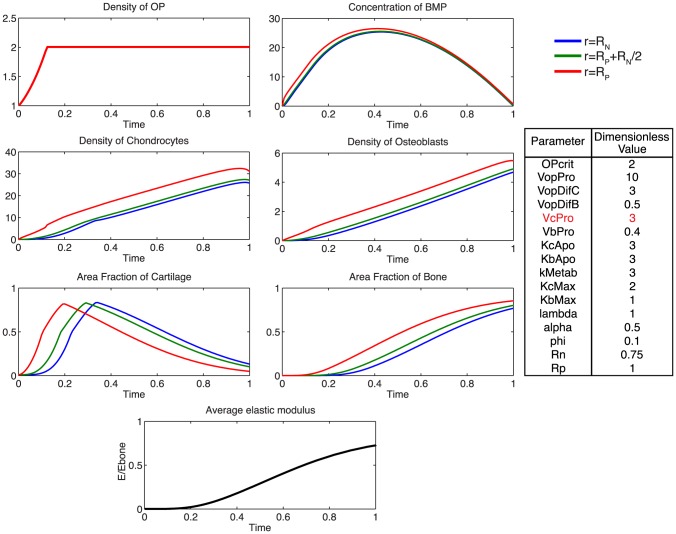
Effect of increasing 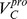. Increasing the rate of chondrocyte proliferation results in a greatly increased density of chondrocytes, and faster rate of consumption of BMP.

**Figure 10:**
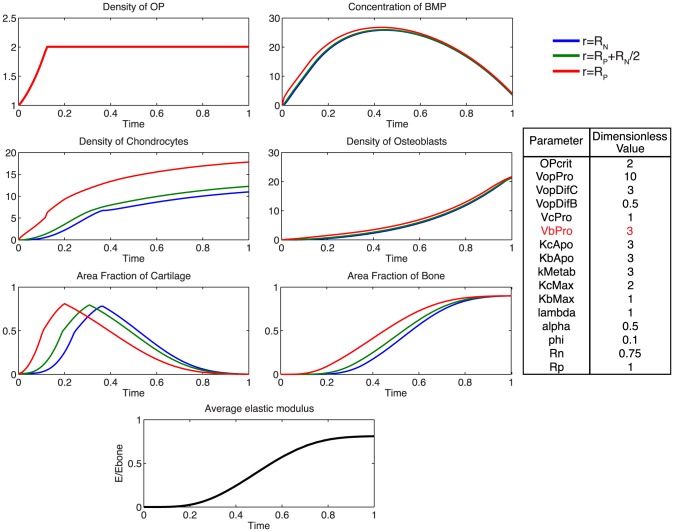
Effect of increasing 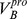. Increasing the rate of osteoblast proliferation results in a greatly increased density of osteoblasts, and more rapid mineralization with a more homogenous distribution of bone tissue at the final time.

**Figure 11:**
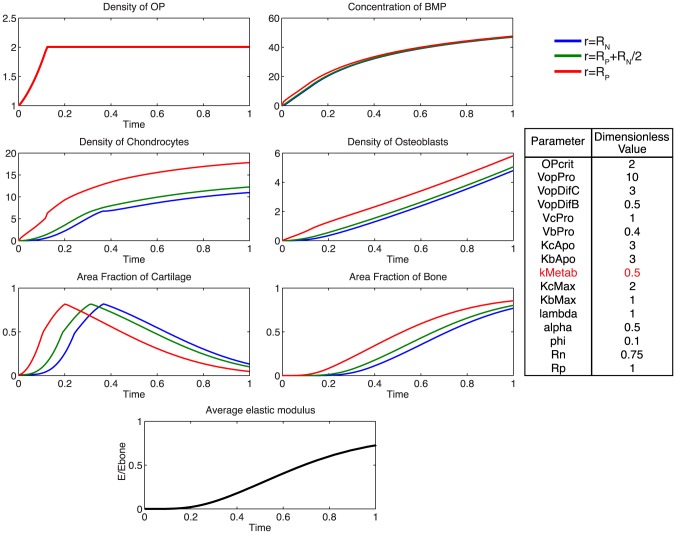
Effect of increasing 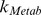. Increasing the rate of consumption of BMP by chondrocytes and osteoblasts results in negative values for BMP, which is not physiologically plausible. A decrease in 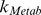 leaves considerably more BMP in the defect space, but does not notably alter ECM production as the processes are likely saturated.

**Figure 12:**
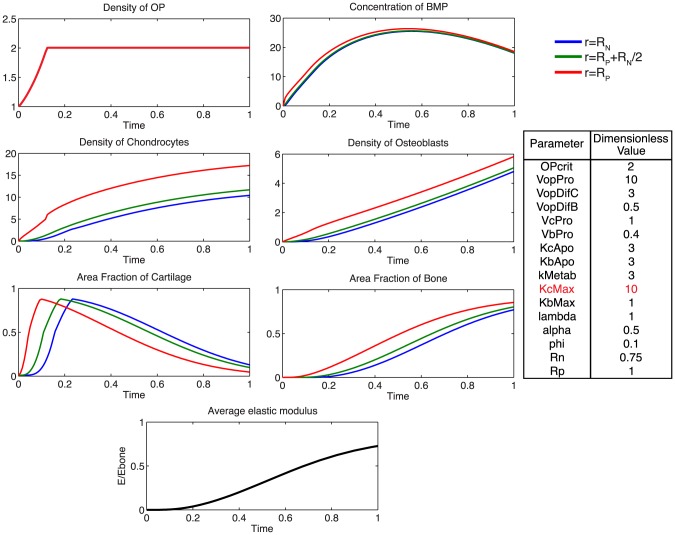
Effect of increasing 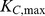. Increasing the maximum rate of cartilage production by chondrocytes, results in a much greater fraction of cartilage at early time points, and a slightly more gradual mineralization process.

Additionally, using the model, we probe the relative effects of key parameters with respect to the ideal healing outcome condition in several biologically relevant scenarios. Increasing the rate of differentiation of osteoprogenitor cells to chondrocytes, 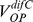, contributes to a more rapid increased density of chondrocytes, as well as more rapid callus formation (Fig. 7). Similarly, increasing the rate of differentiation of osteoprogenitor cells to osteoblasts, 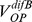, results in an increased density of osteoblasts, slightly decreased density of chondrocytes, as well as a more rapid mineralization of cartilage to bone (Fig. 8). Increasing the rate of proliferation of both chondrocytes (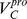) and osteoblasts (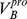) dramatically increases the cell density of each population (Fig. 9, 10). Increasing the rate of consumption of BMP by chondrocytes and osteoblasts (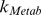) results in negative values for 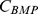, and is therefore not physiologically plausible given the current definition of model parameters. Decreasing 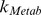 leaves considerably more BMP in the defect space, increasing most notably the density of chondrocytes and production of cartilage (Fig. 11). Increasing the maximum rate of cartilage production by chondrocytes (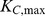) dramatically increases the area fraction of cartilage while simultaneously decreasing the density of chondrocytes as the density of ECM reaches the threshold for apoptosis sooner (Fig. 12).

### Comparing Model Predictions to Two Experimental Cases

*In vivo* experiments harnessing the regenerative capability of the periosteum to infill critical sized defects have been performed in ovine models [1], [4]. Two experiments provide ideal case studies to explore the power of the model to predict potential biological mechanisms leading to observed outcomes. In the first case study, resected autologous periosteal graft is tucked into a periosteal substitute membrane, which is then sutured around the critical sized defect, and stabilized by an intramedullary nail. In the second case study, a patent (intact vascularity) periosteal sleeve is sutured *in situ* after removal of underlying cortical bone and similar placement of an intramedullary nail for mechanical stabilization. The case studies are of particular interest, as they share a common final desired outcome of full tissue generation and healing of the defect at 16 weeks after surgery. However, previous studies indicate that the two case studies each exhibit a distinct time course for tissue generation as well as mechanism of mineralization.

**Figure 13:**
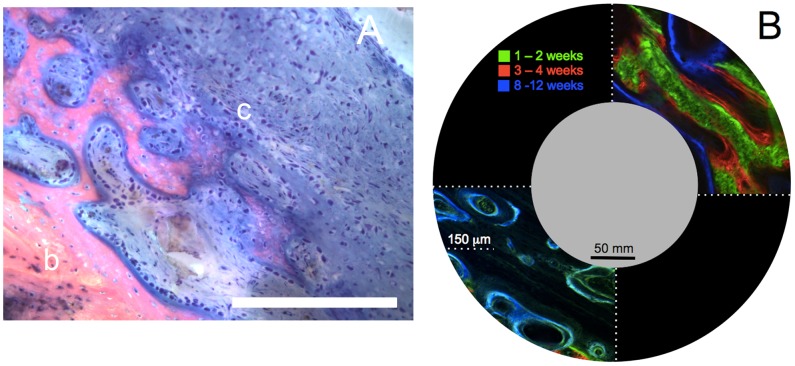
Spatiotemporal assessment of endochondral ossification. Although from different experimental cohorts, the time course of endochondral ossification observed as a gradient of green to red in the right hand case study in (**B**) can be tied to the spatial gradient of mineralization observed as a gradient from pink to blue in (**A**). (**A**) Endochondral ossification of cartilage template (c) to bone (b) by osteoblasts seen as the densely blue-staining rounded cells lining the interface of mineralized tissue and cartilage. Staining of histological specimens enables quantitative assessment of ECM outcome and cell density at a given time in the healing process. Chondrocytes are present in the cartilage matrix, and appear more irregularly shaped. Scale bar = 100 µm. (**B**) Spatial and temporal aspects of defect filling via cellular tissue genesis. Insets in upper right and lower left quadrants (note length scale compared to length scale of defect and IM nail) depict temporal bone formation through visualization of fluorochromes, which chelate to mineral as the ECM is mineralized. The upper right quadrant shows the case study in which patent periosteum is sutured *in situ* around the defect; direct intramembranous bone formation (rapid mineralization of callus) is observed within two weeks (green), and subsequent osteoblastic bone formation (red, blue) occurs via lamellar apposition. The lower left quadrant demonstrates a case in which bone graft is packed in the defect before suturing of patent periosteum around the defect; bone remodeling is observed in the graft-filled defect zone (blue, green) and endochondral bone formation is observed between the underside of the periosteum and the outer edge of the packed bone graft (not shown). *Cf.* [18] for original micrographs showing full field of view.

Healing outcomes are assessed at 16 weeks, where tissue blocks are prepared for hard tissue histology, including Giemsa-eosin staining and fluorochrome microscopy. Giemsa-eosin staining dyes cartilage and cell nuclei blue, and mineralized bone tissue pink (Fig. 13A), offering an ideal comparison between model parameters and biological outcomes at a given time point. The nature of histological staining, however, does not enable temporal analysis of key variables as tissue must be fixed and processed. The chelation of fluorochromes, administered at distinct time-points (*e.g.* 2 weeks, 4 weeks) enables a semi-quantitative assessment of the extent of mineralization, where unique fluorescence wavelengths are utilized to indicate mineralization occurring during a known time span (Fig. 13B).

Comparing final outcome measures between the two experimental case studies, a larger area (in cross section, volume in full tissue block) of callus generation was observed when periosteum graft is incorporated in a periosteum substitute implant than when periosteum is sutured around the defect *in situ.* From micro-computed tomography (μCT) of the entire callus regenerated via periosteum sutured *in situ*, callus volume comprised 3500 mm^3^ out of the 4000 mm^3^, or 87.5% of total defect space [1], with cross-sectional area of tissue regenerate measured in histological cross sections proportional to representative volume. The μCT-measured volume corresponds well to the computational model parameter phi value of 0.1, corresponding to 90% callus infilling. Based on μCT measures of the case study in which periosteum is sutured *in situ* around the defect, total bone volume comprises approximately 40% of callus tissue regenerate. In contrast, periosteum mediated bone generation in the case where the periosteum substitute is used results in approximately 60% filling of the defect with bone; in this case study, quantitative μCT measures could not be made due to retention of the IM nail which leads to imaging artifacts. Though of the same order of magnitude, differences in bone generation between the two case studies may be attributed to differences in tissue regenerate composition, which result from parameters including relative cell populations, as well as differentiation and proliferation rates.

**Figure 14:**
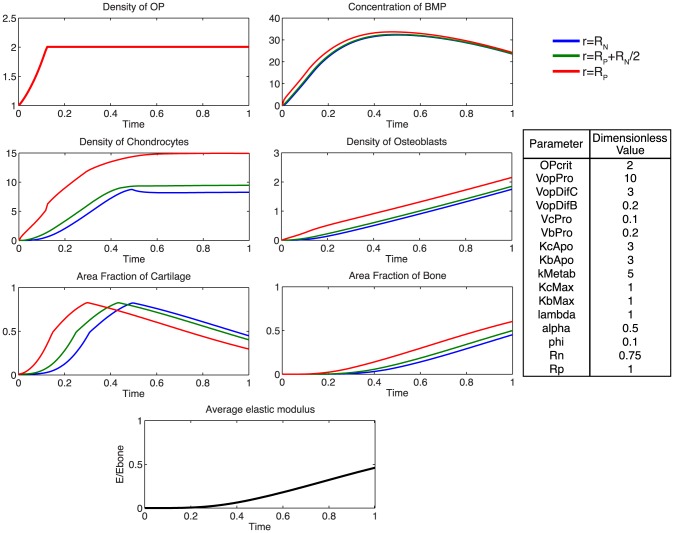
Parametric elucidation of callus healing at 16 weeks in ovine models. Simulation of observed outcomes in two experimental cases where a critical sized femoral defect is enveloped by periosteum or a periosteum substitute.

To begin to elucidate which predictive model parameters may lead to these observed differences in outcomes, model parameters are varied parametrically to achieve experimentally relevant ECM area outcomes. As an initial approach midway between the two experimental case study outcomes, ECM area outcomes were targeted at 50% bone and 50% cartilage comprising the total, final callus cross-section (Fig. 14). To achieve the experimentally relevant outcomes from the complete set of parameters of relevance for healing, the rate of differentiation of osteoprogenitor cells to osteoblasts, as well as the proliferation rate of chondrocytes and osteoblasts must be reduced. Additionally, cartilage and bone are formed at the same rate, whereas complete healing outcome analyzed previously (Fig. 5) requires a faster rate of cartilage production from chondrocytes.

**Figure 15:**
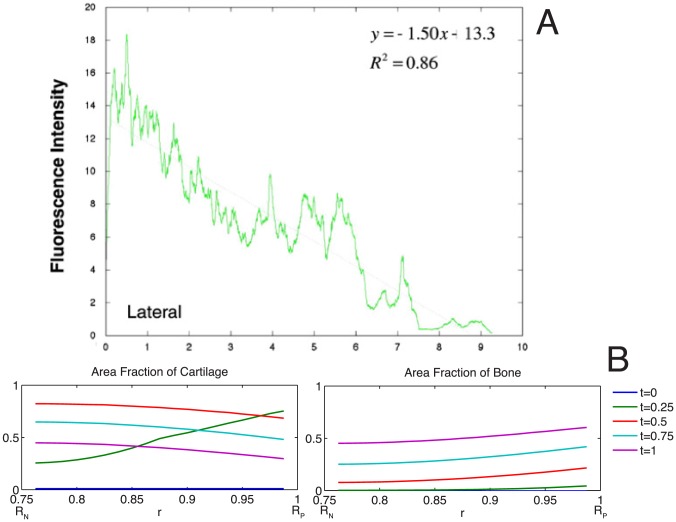
Comparison of model-predicted spatial profiles with experimental measurements. (**A**) Experimental result: radial distribution of mineralization from the periosteum (0) to the intramedullary nail (10) at 2 weeks indicates more bone formation adjacent to the periosteum, with mineralization/chelation significant correlated to radius, and little to no bone present at the intramedullary nail. [18] (**B**) Model predictions at early times (for a 16-week experiment, t = 0.25 corresponds to 4 weeks) indicate a similar distribution of bone, with no mineralized tissue at the surface of the nail.

Histological experimental measures including fluorescence intensity of the fluorochrome administered after two weeks of healing are comparable with computational predictions. Specifically, the radial intensity of the chelated fluorochrome, a measure of chelated fluorochrome and thus mineral concentration, significant correlates to periosteal proximity, where mineral concentration increases with increasing proximity to the periosteum and distance from the IM nail [18]. These data match the predicted gradients in BMP, cells and tissue fractions over time, as predicted by the computational model (Fig. 15). Taken together, the data from these two case studies demonstrate the feasibility of the predictive model.

## Discussion

In the preceding we demonstrate the development of a novel model framework, including cellular, mechanical and biochemical factors, and dynamics of tissue genesis. The mechanistic model that pairs FE mechanics and cellular-tissue dynamics successfully predicts effects of each rate process contributing to endochondral bone formation in postnatal critical sized bone defects, as observed in data from a series of experimental studies using a common ovine defect model. Together with data from experiments using the one-stage bone transport and periosteum substitutes, the model framework provides a novel means to elucidate the inherently complex process of *in vivo*, postnatal bone neogenesis in tissue defects.

The initial outcomes of the model motivate study of the mechano-regulatory process of progenitor cells to explain key spatial and temporal aspect of bone regeneration, resulting here in a simple model framework for testing mechanobiological hypotheses. Taken as a whole, the one-stage bone transport model studies present an interesting new clinical approach to promote healing via periosteally mediated bone regeneration *in situ*. Additionally, the one-stage bone transport model provides a clinically relevant lens from which to focus on modeling the biomechanical processes of bone regeneration in a critical sized defect covered by periosteum. Interestingly, the experimental model offers intrinsic advantages with regard to defining the boundary conditions of the computational model. For example, by virtue of the IM nail, periosteum (or substitute), and proximodistal bone at defect edges (1.27 cm from the defect center), the defect boundary conditions are uniquely defined. In addition, as it defines the outer boundary of the defect and the medullary niche (a source of MSCs) is completely filled by the IM nail, periosteum is the primary source of progenitor cells during defect healing.

While the initial outcomes of the integrated model compare well to *in vivo* large animal regeneration outcomes, experimental determination of key parameters will enable more accurate and complete model predictions. The importance of mechanical modulation of factors such as BMP is highlighted as a key regulator of cellular processes, in particular proliferation and differentiation rates, capable of predicting trends in defect infilling. However, it should be noted that in this first generation model, BMP represents a class of factors that my coordinate tissue genesis and bone healing.

Designed and tested for its capacity to predict observed outcomes in an experimental model with well characterized initial and boundary conditions as well as endpoints, next generations of the model can be refined to test other chemical factors or mechanical scenarios in the future. Specifically, increased sophistication with regard to several key idealizations will make future generations of the model more physiological and will potentially increase its predictive value. For instance from an anatomical perspective, cancellous bone was not accounted for in the mechanical model; while this idealization may be appropriate in consideration of its effect on strain at the middiaphysis, it limits the application of the model to cortical bone defects and ignores the metaphyseal compartment as a potential longer range source of progenitor cells. Furthermore, the model current addresses the process of bone formation via endochondral ossification alone, while it is known that osteogenesis can also proceed directly via an intramembranous pathway [1]. Finally, the current model does not incorporate cell motility or cell-cell interactions, which are known to be important mediators of cell signaling as well as modulator of emergent tissue architecture [3], [60].

While the presented model framework is limited by a number of assumptions and simplifications, its utility will be potentiated as our understanding of the complex process of tissue genesis and healing becomes better understood. For example, with increased understanding of cell signaling and cell behavior during tissue genesis, inclusion of additional complexity in the model will allow for testing of hypotheses, prioritization of experiments, and may contribute to a more complete understanding of the mechanically mediated process established here. The multi-scale component of integrating cellular and biochemical processes with tissue-scale mechanics and quantification contributes to a small but growing body of work.

This work additionally underscores the necessity for a deeper quantitative understanding of the basic biological process of bone regeneration. Notably, the biological signal transduction of mechanical environment is not yet well understood in terms of the time scale, magnitude, duration and cascade of growth factors produced in response to specific mechanical stimuli [3]. Immunohistochemistry and biochemical tools such as RT-PCR, Western blotting, and cell sorting will help quantify factor production following a given mechanical stimulus, in particular as these processes begin to be elucidated in progenitor cells from human periosteum [61]. Additionally, the effect of growth factor concentration on the relative rates of cellular differentiation and proliferation, and the extent to which spatial and temporal presentation alter pathways is an interesting area of study in context of future model development. Many growth factors are involved in the process of cartilage and bone tissue regeneration [62], and understanding their relative and synergistic contributions will be vital to improving model predictions.

Measurement of the inherent delay between triggering of cellular processes such as mechanotransduction resulting in up- or down-regulation of gene transcription, as well as ECM protein secretion and posttranslational modification may also contribute to estimating actual values of proliferation and differentiation rates, and should be assessed in future studies. Additionally, the rate of production of ECM components is one of several specific factors implicated in triggering rapid formation of structural tissue from cells, as well as their rate-limiting processes. From a therapeutic perspective, speeding the formation of a cartilage template and triggering a temporal increase in osteoblast density may help speed bridging time. Direct intramembranous bone formation, an endogenous means for rapid repair [1], [3], is a further natural paradigm that would lend itself well for study with the current model framework.

Further inherent limitations of the current model relate to the number of idealizations necessary to build and test the feasibility of the initial model platform. Next generation, follow on models may also incorporate additional biological and mechanical factors known to alter tissue regeneration in healing defects. Notably, the magnitude and duration of deviatoric and dilatational mechanical signals are known to modulate proliferation and differentiation pathways [3], [63]. Additionally, the early formation of vascular supply is implicated as playing an important role in regeneration [64], where the role of oxygen tension alters chondro- and osteogenesis in the healing callus [65], [66]. Models that describe the relationship between angiogenesis and bone regeneration have been previously established [29], [67] and may be readily incorporated into the mechanistic model framework presented here. Finally, the explicit depiction of cell motility as well as cell apoptosis in future models will add a further dynamic aspect that may better account for inherent differences in bone tissue genesis via intramembranous and endochondral mechanisms, which themselves represent variations on tissue genesis algorithms via epithelial to mesenchymal and mesenchymal to epithelial transitions [3].

Future versions of tissue genesis models may also integrate the mechanical model to provide a real-time strain stimulus, rather than a fitted-relationship value. This integration will allow for the analysis of the effects of dynamic loading conditions such as walking versus running, or therapeutic treatments to optimize stimulus for maximum quantity and quality of tissue regeneration. Individual-specific anatomic data may also be integrated into the mechanical model simulation to assess injury-specific regimens.

Looking forward to the next generation of periosteal implants and tissue-engineered replacements, specific application tissue healing may be modeled to test *in silico*, thereby providing a high-throughput test for critical parameters. More complex models may assess the material properties of the periosteum substrate in context of transmitting mechanical cues to underlying progenitor cells, or from a poroelastic and permeability perspective to guide nutrients into the defect space [4], [68], [69].

In conclusion, the model framework presented here offers a novel integration of a mechanistic feedback system based on the mechanosensitivity of periosteal progenitor cells to model and predict tissue regeneration on multiple length and time scales. The complex process of *de novo* bone regeneration involves many additional cellular and biochemical processes that should be incorporated in the future to improve the model's applicability. Mechanistic models offer great potential to both clinicians and researchers hoping to develop new techniques and insight into the process of bone regeneration, ultimately looking forward to novel therapies to improve patient outcomes.

## Supporting Information

Figure S1 **Dimensionless governing equations.** (**A**) Dimensionless variables are defined *per* the following equations. (**B**) Dimensionless parameters are defined *per* the following equations, and include (**C**) osteoprogenitor cells, (**D**) bone morphogenetic protein, (**E**) mechanical factors, (**F**) chondrocytes, (**G**) osteoblasts, and (**H**) production of extracellular matrix.
(DOCX)

Figure S2 **Discretized dimensionless governing equations using method of lines and accounting for (A) spatial discretization over the domain, (B) osteoprogenitor cells, and their relevant boundary condition, (C) bone morphogenetic protein (BMP) and the relevant boundary condition for BMP dynamics, (D) mechanical relationships, (E) chondrocytes, (F) osteoblasts, and (G) extracellular matrix production.**
(DOCX)
