# Supplementary material for: Mechanistic, Mathematical Model to Predict the Dynamics of Tissue Genesis in Bone Defects via Mechanical Feedback and Mediation of Biochemical Factors
Source: PLoS Comput Biol. 2014 Jun 26;10(6):e1003604. doi: 10.1371/journal.pcbi.1003604 (PMC4072518; doi:10.1371/journal.pcbi.1003604)
Supplement: Figure S1 — Dimensionless governing equations. (A) Dimensionless variables are defined per the following equations. (B) Dimensionless parameters are defined per the following equations, and include (C) osteoprogenitor cells, (D) bone morphogenetic protein, (E) mechanical factors, (F) chondrocytes, (G) osteoblasts, and (H) production of extracellular matrix. (DOCX) [file pcbi.1003604.s001.docx]

**A**

$$\begin{matrix} \bar{\Omega}_{OP}=\frac{\Omega_{OP}}{\Omega_{OP}^{0}\text{ }},\text{ }\bar{\Omega}_{C}\text{=}\frac{R_{P}\Omega_{C}}{\Omega_{OP}^{0}\text{ }},\text{ }\bar{\Omega}_{B}\text{=}\frac{R_{P}\Omega_{B}}{\Omega_{OP}^{0}\text{ }},\text{ }\bar{C}_{BMP}=\frac{R_{P}C_{BMP}}{\Omega_{OP}^{0}\text{ }} \\ \tau=tK_{B,\max},\text{ }\eta\text{=}\frac{r}{R_{P}},\text{ }\bar{E}(\eta,\tau)=\frac{E(r,t)}{E_{B}},\text{ }\bar{E}_{ave}(\tau)=\frac{E_{ave}(t)}{E_{B}} \\ \text{ }\bar{k}_{OP}^{pro}=\frac{k_{OP}^{pro}}{K_{B,\max}},\text{ }\bar{k}_{C}^{pro}=\frac{k_{C}^{pro}}{K_{B,\max}},\text{ }\bar{k}_{B}^{pro}=\frac{k_{B}^{pro}}{K_{B,\max}}\text{ } \\ \overline{k}_{OP}^{difC}=\frac{k_{OP}^{difC}}{K_{B,\max}},\text{ }\overline{k}_{OP}^{difB}=\frac{k_{OP}^{difB}}{K_{B,\max}} \\ \overline{k}_{C}^{apo}=\frac{k_{C}^{apo}}{K_{B,\max}},\text{ }\overline{k}_{B}^{apo}=\frac{k_{B}^{apo}}{K_{B,\max}} \\ \overline{K}_{C}=\frac{K_{C}\Omega_{OP}^{0}}{R_{P}K_{B,\max}},\text{ }\overline{K}_{B}=\frac{K_{B}}{K_{B,\max}} \end{matrix}$$

**B**

**C**

$$\frac{d\Omega_{OP}}{dt}=\left[k_{OP}^{pro}(t)-k_{OP}^{difC}(t)-k_{OP}^{difB}\right]\Omega_{OP};\text{ }\Omega_{OP}\left(0\right)=\Omega_{OP}^{0}\text{ }$$

$$\frac{d\bar{\Omega}_{OP}}{d\tau}=\left[\bar{k}_{OP}^{pro}(\tau)-\bar{k}_{OP}^{difC}(\tau)-\overline{k}_{OP}^{difB}\right]\bar{\Omega}_{OP};\text{ }\bar{\Omega}_{OP}\left(0\right)=1$$

$$k_{OP}^{pro}(t)=\left\{\begin{matrix} \frac{V_{OP}^{pro}C_{BMP}(R_{p},t)}{K_{OP}^{pro}+C_{BMP}(R_{p},t)},\text{ }\Omega_{OP}<\Omega_{OP}^{crit} \\ k_{OP}^{difC}(t)+k_{OP}^{difB}(t),\text{ }\Omega_{OP}\geq\Omega_{OP}^{crit} \end{matrix}\right\}$$

$$\overline{k}_{OP}^{pro}(\tau)=\left\{\begin{matrix} \frac{\overline{V}_{OP}^{pro}\overline{C}_{BMP}(1,\tau)}{\overline{K}_{OP}^{pro}+\overline{C}_{BMP}(1,\tau)},\text{ }\overline{\Omega}_{OP}<\overline{\Omega}_{OP}^{crit} \\ \overline{k}_{OP}^{difC}(\tau)+\overline{k}_{OP}^{difB}(\tau),\text{ }\overline{\Omega}_{OP}\geq\overline{\Omega}_{OP}^{crit} \end{matrix}\right\}$$

$k_{OP}^{difC}(t)=\frac{V_{OP}^{difC}C_{BMP}(R_{p},t)}{K_{OP}^{difC}+C_{BMP}(R_{p},t)}$ $\overline{k}_{OP}^{difC}(\tau)=\frac{\overline{V}_{OP}^{difC}\overline{C}_{BMP}(1,\tau)}{\overline{K}_{OP}^{difC}+\overline{C}_{BMP}(1,\tau)}$

$k_{OP}^{difB}(t)=\frac{V_{OP}^{difB}C_{BMP}(R_{p},t)}{K_{OP}^{difB}+C_{BMP}(R_{p},t)}$ $\overline{k}_{OP}^{difB}(\tau)=\frac{\overline{V}_{OP}^{difB}\overline{C}_{BMP}(1,\tau)}{\overline{K}_{OP}^{difB}+\overline{C}_{BMP}(1,\tau)}$

**D**

$$\frac{\partial C_{BMP}}{\partial t}=D_{BMP}\left[\frac{\partial^{2}C_{BMP}}{\partial r^{2}}+\frac{1}{r}\frac{\partial C_{BMP}}{\partial r}\right]-k_{Metab}(\Omega_{C}+\Omega_{B}),\text{ }R_{N}<r<R_{P}$$

$$\text{ }\frac{\partial\overline{C}_{BMP}}{\partial\tau}=\overline{D}_{BMP}\left[\frac{\partial^{2}\overline{C}_{BMP}}{\partial\eta^{2}}+\frac{1}{\eta}\frac{\partial\overline{C}_{BMP}}{\partial\eta}\right]-\overline{k}_{Metab}(\overline{\Omega}_{C}+\overline{\Omega}_{B}),\text{ }\frac{R_{N}}{R_{P}}<\eta<1$$

$C_{BMP}(r,0)=0$ $\overline{C}_{BMP}(\eta,0)=0$

$r=R_{N}:\text{ }\frac{\partial C_{BMP}}{\partial r}=0$ $\eta=\frac{R_{N}}{R_{P}}:\text{ }\frac{\partial\overline{C}_{BMP}}{\partial\eta}=0$

$r=R_{P}:\text{ }D_{BMP}\frac{\partial C_{BMP}}{\partial r}=k_{Mech}\varepsilon_{N}(t)\Omega_{OP}$ $\eta=1:\text{ }\frac{\partial\overline{C}_{BMP}}{\partial\eta}=\overline{k}_{Mech}\varepsilon_{N}(\tau)\overline{\Omega}_{OP}$

**E**

$$\varepsilon_{N}(t)=F[E_{ave}(t)],\text{ }\varepsilon_{N}(\tau)=F[\bar{E}_{ave}(\tau)]$$

$E_{ave}(t)=\frac{2}{(R_{p}^{2}-R_{N}^{2})}\int_{R_{N}}^{R_{P}} E(r,t)rdr\text{ }$ $\overline{E}_{ave}(\tau)=\frac{2}{1-\left(R_{N}/R_{P}\right)^{2}}\int_{R_{N}/R_{P}}^{1} \overline{E}(\eta,\tau)\eta d\eta$

$E(r,t)=A_{C}(r,t)E_{C}+A_{B}(r,t)E_{B}$ $\overline{E}(\eta,\tau)=A_{C}(\eta,\tau)\overline{E}_{C/B}+A_{B}(\eta,\tau)$

$$\varepsilon_{N}(\tau)=-1.458\ln\left(E_{B}\overline{E}_{ave}(\tau)\right)+5.788=-1.458\ln(E_{B})\ln\left(\overline{E}_{ave}(\tau)\right)+5.788$$

**F**

$$\text{ }\frac{\partial\Omega_{C}}{\partial t}=\mu_{C}\left[\frac{\partial^{2}\Omega_{C}}{\partial r^{2}}+\frac{1}{r}\frac{\partial\Omega_{C}}{\partial r}\right]+\left[k_{C}^{pro}(r,t)-k_{C}^{apo}(r,t)\right]\Omega_{C},\text{ R}_{N}<r<\text{R}_{P}\text{ }$$

$$\frac{\partial\overline{\Omega}_{C}}{\partial\tau}=\overline{\mu}_{C}\left[\frac{\partial^{2}\overline{\Omega}_{C}}{\partial\eta^{2}}+\frac{1}{\eta}\frac{\partial\overline{\Omega}_{C}}{\partial\eta}\right]+\left[\overline{k}_{C}^{pro}(\eta,\tau)-\overline{k}_{C}^{apo}(\eta,\tau)\right]\overline{\Omega}_{C},\text{ }\frac{R_{N}}{R_{P}}<\eta<1$$

$k_{C}^{pro}(r,t)=\frac{V_{C}^{pro}C_{BMP}(r,t)}{K_{C}^{pro}+C_{BMP}(r,t)}$ $\overline{k}_{C}^{pro}(\eta,\tau)=\frac{\overline{V}_{C}^{pro}\overline{C}_{BMP}(\eta,\tau)}{\overline{K}_{C}^{pro}+\overline{C}_{BMP}(\eta,\tau)}$

$r=R_{N}:\text{ }\frac{\partial\Omega_{C}}{\partial r}=0$ $\eta=\frac{R_{N}}{R_{P}}:\text{ }\frac{\partial\overline{\Omega}_{C}}{\partial\eta}=0$

$$r=R_{P}:\text{ }\mu_{C}\frac{\partial\Omega_{C}}{\partial r}=k_{OP}^{difC}\Omega_{OP}$$

$$\eta=1:\text{ }\frac{\partial\bar{\Omega}_{C}}{\partial\eta}=\frac{\overline{k}_{OP}^{difC}}{\overline{\mu}_{C}}\overline{\Omega}_{OP}$$

$\overline{\Omega}_{C}(\eta,0)=0$

**G**

$$\text{ }\frac{\partial\Omega_{B}}{\partial t}=\mu_{B}\left[\frac{\partial^{2}\Omega_{B}}{\partial r^{2}}+\frac{1}{r}\frac{\partial\Omega_{B}}{\partial r}\right]+\left[k_{B}^{pro}(r,t)-k_{B}^{apo}(r,t)\right]\Omega_{B},\text{ R}_{N}<r<\text{R}_{P}\text{ }$$

$$\frac{\partial\overline{\Omega}_{B}}{\partial\tau}=\overline{\mu}_{B}\left[\frac{\partial^{2}\overline{\Omega}_{B}}{\partial\eta^{2}}+\frac{1}{\eta}\frac{\partial\overline{\Omega}_{B}}{\partial\eta}\right]+\left[\overline{k}_{B}^{pro}(\eta,\tau)-\overline{k}_{B}^{apo}(\eta,\tau)\right]\overline{\Omega}_{B},\text{ }\frac{R_{N}}{R_{P}}<\eta<1$$

$k_{B}^{pro}(r,t)=\frac{V_{B}^{pro}C_{BMP}(r,t)}{K_{B}^{pro}+C_{BMP}(r,t)}$ $\overline{k}_{B}^{pro}(\eta,\tau)=\frac{\overline{V}_{B}^{pro}\overline{C}_{BMP}(\eta,\tau)}{\overline{K}_{B}^{pro}+\overline{C}_{BMP}(\eta,\tau)}$

$$k_{B}^{apo}(r,t)=K_{B}^{apo}A_{B}\left\{\begin{matrix} 0,\text{ }A_{B}<A_{B,\max} \\ 1,\text{ }A_{B}\geq A_{B,\max} \end{matrix}\right\}$$

$$\overline{k}_{B}^{apo}(\eta,\tau)=\overline{K}_{B}^{apo}A_{B}\left\{\begin{matrix} 0,\text{ }A_{B}<A_{B,\max} \\ 1,\text{ }A_{B}\geq A_{B,\max} \end{matrix}\right\}$$

$$r=R_{N}:\text{ }\frac{\partial\Omega_{B}}{\partial r}=0$$

$$\eta=\frac{R_{N}}{R_{P}}:\text{ }\frac{\partial\overline{\Omega}_{B}}{\partial\eta}=0$$

$$r=R_{P}:\text{ }\mu_{B}\frac{\partial\Omega_{B}}{\partial r}=k_{OP}^{difB}(R_{P},t)\Omega_{OP}$$

$$\eta=1:\text{ }\frac{\partial\bar{\Omega}_{B}}{\partial\eta}=\frac{\overline{k}_{OP}^{difB}}{\overline{\mu}_{B}}\overline{\Omega}_{OP}$$

$\Omega_{B}(r,0)=0$ $\overline{\Omega}_{B}(\eta,0)=0$

**H**

, where and $\frac{R_{N}}{R_{P}}<\eta<1$

$\frac{\partial A_{C}}{\partial t}=K_{C}\Omega_{C}-K_{B}A_{C};\text{ }A_{C}(0)=0$ $\frac{\partial A_{C}}{\partial\tau}=\overline{K}_{C}\overline{\Omega}_{C}-\overline{K}_{B}A_{C};\text{ }A_{C}(0)=0$

$\frac{\partial A_{B}}{\partial t}=K_{B}A_{C};\text{ }A_{B}(0)=0$ $\frac{\partial A_{B}}{\partial\tau}=\overline{K}_{B}A_{C};\text{ }A_{B}(0)=0$

$K_{B}=K_{B,\max}\Omega_{B}$ $\overline{K}_{B}=\overline{K}_{B,\max}\overline{\Omega}_{B}$
