# Supplementary material for: Mechanistic, Mathematical Model to Predict the Dynamics of Tissue Genesis in Bone Defects via Mechanical Feedback and Mediation of Biochemical Factors
Source: PLoS Comput Biol. 2014 Jun 26;10(6):e1003604. doi: 10.1371/journal.pcbi.1003604 (PMC4072518; doi:10.1371/journal.pcbi.1003604)
Supplement: Figure S2 — Discretized dimensionless governing equations using method of lines and accounting for (A) spatial discretization over the domain, (B) osteoprogenitor cells, and their relevant boundary condition, (C) bone morphogenetic protein (BMP) and the relevant boundary condition for BMP dynamics, (D) mechanical relationships, (E) chondrocytes, (F) osteoblasts, and (G) extracellular matrix production. (DOCX) [file pcbi.1003604.s002.docx]

**A**

$$\begin{matrix} R_{N}/R_{P}\leq\eta\leq1\text{ }\Rightarrow\text{ i=0,1,2,...N } \\ \Delta=\frac{1-R_{N}/R_{P}}{N}\text{ }\eta_{i}=i\Delta+R_{N}/R_{P} \end{matrix}$$

**B**

Osteoprogenitor cells are located at the periosteum, (i=N)

$$\frac{d\overline{\Omega}_{OP}}{d\tau}=\left[\overline{k}_{OP}^{pro}-\overline{k}_{OP}^{difC}-k_{OP}^{difB}\right]\overline{\Omega}_{OP};\text{ }\overline{\Omega}_{OP}(0)=1$$

$\overline{k}_{OP}^{pro}=\left\{\begin{matrix} \frac{\overline{V}_{OP}^{pro}\overline{C}_{BMP}(N)}{\overline{K}_{OP}^{pro}+\overline{C}_{BMP}(N)},\text{ }\overline{\Omega}_{OP}<\overline{\Omega}_{OP}^{crit} \\ \overline{k}_{OP}^{difC}+\overline{k}_{OP}^{difB},\text{ }\overline{\Omega}_{OP}\geq\overline{\Omega}_{OP}^{crit} \end{matrix}\right\}$ $\overline{k}_{OP}^{difC}=\frac{\overline{V}_{OP}^{difC}\overline{C}_{BMP}(N)}{\overline{K}_{OP}^{difC}+\overline{C}_{BMP}(N)}$

$$\overline{k}_{OP}^{difB}=\frac{\overline{V}_{OP}^{difB}\overline{C}_{BMP}(N)}{\overline{K}_{OP}^{difB}+\overline{C}_{BMP}(N)}$$

**C**

The BMP dynamics are specified within the defect boundaries: $i=1,2...N-1$

$$\begin{matrix} \frac{d\overline{C}_{BMP}(i)}{d\tau}=\overline{D}_{BMP}\left[\frac{\overline{C}_{BMP}(i+1)-2\overline{C}_{BMP}(i)+\overline{C}_{BMP}(i-1)}{\Delta^{2}}+\frac{1}{i\Delta+R_{N}/R_{P}}\frac{\overline{C}_{BMP}(i)-\overline{C}_{BMP}(i-1)}{\Delta}\right] \\ -\overline{k}_{Metab}\left[\overline{\Omega}_{C}(i)+\Omega_{B}(i)\right] \end{matrix}$$

IC: $\overline{C}_{BMP}(i,0)=0$

At the boundaries of the defect:

$i=0:\text{ }\overline{C}_{BMP}(0)=\overline{C}_{BMP}(1)$ $\text{ }i=N:\text{ }\overline{C}_{BMP}(N)=\overline{C}_{BMP}(N-1)+\Delta\overline{k}_{Mech}\varepsilon_{N}\overline{\Omega}_{OP}$

Substitute the boundary conditions for i=1 and i=N-1 to get

$$\frac{d\overline{C}_{BMP}(1)}{d\tau}=\overline{D}_{BMP}\left[\frac{\overline{C}_{BMP}(2)-\overline{C}_{BMP}(1)}{\Delta^{2}}\right]-\overline{k}_{Metab}\left[\overline{\Omega}_{C}(1)+\Omega_{B}(1)\right]$$

$$\begin{matrix} \frac{d\overline{C}_{BMP}(N-1)}{d\tau}=\overline{D}_{BMP}\left[\frac{\Delta\overline{k}_{Mech}\varepsilon_{N}\overline{\Omega}_{OP}-\overline{C}_{BMP}(N-1)+\overline{C}_{BMP}(N-2)}{\Delta^{2}}+\frac{\overline{C}_{BMP}(N-1)-\overline{C}_{BMP}(N-2)}{\Delta[(N-1)\Delta+R_{N}/R_{P}]}\right] \\ -\overline{k}_{Metab}\left[\overline{\Omega}_{C}(N-1)+\Omega_{B}(N-1)\right] \end{matrix}$$

**D**

$$\varepsilon_{N}(\tau)=F[\bar{E}_{ave}(\tau)]$$

Trapezoid rule for evaluating the definite integral:

$$\overline{E}_{ave}(\tau)=\frac{\Delta}{1-(R_{N}/R_{P})^{2}}\left[f(1,\tau)+2\sum_{i=2}^{N-2} f(i,\tau)+f(N-1,\tau)\right]$$

where

$$f(i,\tau)=\bar{E}(i,\tau)[i\Delta+R_{N}/R_{P}]\text{ (i=1,2,...N-1)}$$

$$\overline{E}(i,\tau)=A_{C}(i,\tau)\overline{E}_{C/B}+A_{B}(i,\tau)$$

**E**

The chondrocyte dynamics are specified within the defect boundaries: $i=1,2...N-1$

$$\frac{d\overline{\Omega}_{C}(i)}{d\tau}=\overline{\mu}_{C}\left[\frac{\overline{\Omega}_{C}(i+1)-2\overline{\Omega}_{C}(i)+\overline{\Omega}_{C}(i-1)}{\Delta^{2}}+\frac{\overline{\Omega}_{C}(i)-\overline{\Omega}_{C}(i-1)}{[i\Delta+R_{N}/R_{P}]\Delta}\right]+\left[\overline{k}_{C}^{pro}(i)-\overline{k}_{C}^{apo}(i)\right]\overline{\Omega}_{C}(i)$$

$$\overline{k}_{C}^{pro}(i)=\frac{\overline{V}_{C}^{pro}\overline{C}_{BMP}(i)}{\overline{K}_{C}^{pro}+\overline{C}_{BMP}(i)}$$

IC: $\overline{\Omega}_{C}(i,0)=0$

At the boundaries of the defect:

$$i=0:\text{ }\overline{\Omega}_{C}(0)=\overline{\Omega}_{C}(1)\text{ }i=N :\text{ }\overline{\Omega}_{C}(N)=\overline{\Omega}_{C}(N-1)+\frac{\Delta\overline{k}_{OP}^{difC}}{\overline{\mu}_{C}}\overline{\Omega}_{OP}$$

Substitute the boundary conditions for i=1 and i=N-1 to get:

$$i=1\text{ }\frac{d\overline{\Omega}_{C}(1)}{d\tau}=\overline{\mu}_{C}\left[\frac{\overline{\Omega}_{C}(2)-\overline{\Omega}_{C}(1)}{\Delta^{2}}\right]+\left[\overline{k}_{C}^{pro}(1)-\overline{k}_{C}^{apo}(1)\right]\overline{\Omega}_{C}(1)$$

$$\begin{matrix} i=N-1\text{ }\frac{d\overline{\Omega}_{C}(N-1)}{d\tau}=\overline{\mu}_{C}\left[\frac{-\overline{\Omega}_{C}(N-1)+\frac{\Delta\overline{k}_{OP}^{difC}}{\overline{\mu}_{C}}\overline{\Omega}_{OP}+\overline{\Omega}_{C}(N-2)}{\Delta^{2}}\right]+\overline{\mu}_{C}\left[\frac{\overline{\Omega}_{C}(N-1)-\overline{\Omega}_{C}(N-2)}{[(N-1)\Delta+R_{N}/R_{P}]\Delta}\right] \\ \text{ }+\left[\overline{k}_{C}^{pro}(N-1)-\overline{k}_{C}^{apo}(N-1)\right]\overline{\Omega}_{C}(N-1) \end{matrix}$$

**F**

The chondrocyte dynamics are specified within the defect boundaries: $i=1,2...N-1$

$$\frac{d\overline{\Omega}_{B}(i)}{d\tau}=\overline{\mu}_{B}\left[\frac{\overline{\Omega}_{B}(i+1)-2\overline{\Omega}_{B}(i)+\overline{\Omega}_{B}(i-1)}{\Delta^{2}}+\frac{\overline{\Omega}_{B}(i)-\overline{\Omega}_{B}(i-1)}{[i\Delta+R_{N}/R_{P}]\Delta}\right]+\left[\overline{k}_{B}^{pro}(i)-\overline{k}_{B}^{apo}(i)\right]\overline{\Omega}_{B}(i)$$

$$\overline{k}_{B}^{pro}(i)=\frac{\overline{V}_{B}^{pro}\overline{C}_{BMP}(i)}{\overline{K}_{B}^{pro}+\overline{C}_{BMP}(i)}$$

$$\overline{k}_{B}^{apo}(i)=\overline{K}_{B}^{apo}A_{B}(i)\left\{\begin{matrix} 0,\text{ }A_{B}(i)<A_{B,\max} \\ 1,\text{ }A_{B}(i)\geq A_{B,\max} \end{matrix}\right\}$$

IC: $\overline{\Omega}_{B}(i,0)=0$

At the boundaries of the defect:

$$i=0:\text{ }\overline{\Omega}_{B}(0)=\overline{\Omega}_{B}(1)\text{ }i=N :\text{ }\overline{\Omega}_{B}(N)=\overline{\Omega}_{B}(N-1)+\frac{\Delta\overline{k}_{OP}^{difB}}{\overline{\mu}_{B}}\overline{\Omega}_{OP}$$

Substitute the boundary conditions for i=1 and i=N-1 to get:

$$i=1\text{ }\frac{d\overline{\Omega}_{B}(1)}{d\tau}=\overline{\mu}_{B}\left[\frac{\overline{\Omega}_{B}(2)-\overline{\Omega}_{B}(1)}{\Delta^{2}}\right]+\left[\overline{k}_{B}^{pro}(1)-\overline{k}_{B}^{apo}(1)\right]\overline{\Omega}_{B}(1)$$

$$\begin{matrix} i=N-1\text{ }\frac{d\overline{\Omega}_{B}(N-1)}{d\tau}=\overline{\mu}_{B}\left[\frac{-\overline{\Omega}_{B}(N-1)+\frac{\Delta\overline{k}_{OP}^{difB}}{\overline{\mu}_{B}}\overline{\Omega}_{OP}+\overline{\Omega}_{B}(N-2)}{\Delta^{2}}\right]+\overline{\mu}_{B}\left[\frac{\overline{\Omega}_{B}(N-1)-\overline{\Omega}_{B}(N-2)}{[(N-1)\Delta+R_{N}/R_{P}]\Delta}\right] \\ \text{ }+\left[\overline{k}_{B}^{pro}(N-1)-\overline{k}_{B}^{apo}(N-1)\right]\overline{\Omega}_{B}(N-1) \end{matrix}$$

**G**

The cartilage and bone dynamics are specified within the defect boundaries:

$$\begin{matrix} \text{ }\frac{dA_{B}(i)}{d\tau}=\overline{K}_{B}(i)A_{C}(i) \\ IC:\text{ }A_{B}(i,0)=0 \\ \\ \overline{K}_{B}(i)=\overline{K}_{B,\max}\overline{\Omega}_{B}(i) \end{matrix}$$
